# Egg Quality and Laying Performance of Rhode Island Red Hens Fed with Black Soldier Fly Larvae and Microalgae Meal as an Alternative Diet

**DOI:** 10.3390/ani15111540

**Published:** 2025-05-24

**Authors:** Marta Montserrat Tovar-Ramírez, Mónica Vanessa Oviedo-Olvera, Maria Isabel Nieto-Ramirez, Benito Parra-Pacheco, Ana Angelica Feregrino-Pérez, Juan Fernando Garcia-Trejo

**Affiliations:** 1 División de Investigación y Posgrado, Facultad de Ingeniería, Universidad Autónoma de Querétaro, Carretera a Chichimequillas Km. 1 s/n Amazcala, El Marques, Querétaro 76265, Mexico; montsemmtr@gmail.com (M.M.T.-R.); moviedo30@alumnos.uaq.mx (M.V.O.-O.); isabel.nieto@uaq.mx (M.I.N.-R.); benito.parra@uaq.mx (B.P.-P.); 2 Cuerpo Académico de Bioingeniería Básica y Aplicada, Facultad de Ingeniería, Universidad Autónoma de Querétaro, Cerro de las Campanas s/n. Las Campanas, Querétaro 76010, Mexico; geli@uaq.mx

**Keywords:** laying-hen eggs, alternative proteins, black soldier fly larvae, microalgae

## Abstract

The potential of black soldier fly larvae (BSFL) and microalgae (MA) in poultry diets has garnered increasing interest due to their high nutritional value and reduced environmental footprint. BSFL represent a sustainable alternative to conventional protein sources such as soybean meal, whereas MA contributes to improved egg quality, particularly through its enrichment with polyunsaturated fatty acids. This study assessed the effects of BSFL and MA inclusion on the growth performance and egg quality of Rhode Island Red (RIR) laying hens. Three diets were formulated: Diet A (10% BSFL), Diet B (10% BSFL + 2% MA), and Diet C (commercial control). The diets were formulated to meet the age-specific nutrient requirements of RIR hens, according to the National Research Council (NRC, 1994) guidelines. A total of 96 four-week-old chicks were randomly allocated to six pens (*n* = 16 per pen) and provided ad libitum access to feed and water throughout the trial. The results demonstrated that the inclusion of BSFL and MA significantly influenced the growth rate, onset of lay, and egg characteristics. Hens fed Diet B exhibited the highest average weekly body weight gain (0.034 ± 0.001 kg/week); initiated laying at 20 weeks of age, three weeks earlier than hens on Diets B and C; and produced significantly heavier eggs (51.208 ± 0.511 g). Enhanced eggshell quality and yolk pigmentation were also observed. In addition, Diet B enhanced the nutritional profile of the eggs, yielding a higher albumen protein content (76.546 ± 1.382%DM) and lower lipid concentrations (0.451 ± 0.128%DM). These findings underscore the potential of BSFL and MA as functional feed ingredients for improving poultry performance and egg quality in a sustainable production system.

## Simple Summary

Using black soldier fly larvae (BSFL) and microalgae (MA) in poultry diets may enhance egg quality and support hen growth. This study tested diets containing BSFL and MA for Rhode Island Red (RIR) hens. Chickens fed a diet with both BSFL and MA grew faster, began laying earlier, and produced larger, higher-quality eggs compared to the control diets. These results suggest that BSFL and MA could provide a valuable alternative in poultry feed.

## 1. Introduction

The poultry industry is the fastest growing sector of livestock, which is expected to reach 7% by the year 2050 [1]. This growth will increase the demand for ingredients in poultry feed [2,3]. Feeding costs in poultry production represents 70% of the total expenses, of which energy and protein represent 95% [4]. The main sources of energy and protein are corn and soybean meal, respectively; both face challenges for their use such as the requirements for marketing food for humans and competition with other industries like the biofuel industry [5]. Consequently, research into the use of alternative ingredients in the formulation of feed for poultry has been encouraged [6].

Two alternative ingredients with promising results are black soldier fly larvae (BSFL) and microalgae (MA). Biotransformation of organic residues with BSFL is a novel technology [7], which produces high-value compounds containing 35–37% protein, 20–35% lipids, and minerals (3.03% calcium and 0.66% phosphorous) [8,9,10].

Other studies have proposed BSFL as an alternative protein source to replace soybean in poultry feed [11]. In laying hens, the results of studies carried out suggest that a total and partial replacement of soybean meal in feed is possible. Marono, in 2017, studied a total replacement in laying hens that were 24–45 weeks old; the results showed a favorable effect on feeding conversion but a negative effect on the laying rate, feed consumption, and egg size [12]. Secci, in 2018, used a total replacement for laying hens of 21 weeks old and obtained results of yolks that were redder and richer in tocopherol, lutein, and carotene, with an 11% reduction in the cholesterol in yolks compared with hens fed a soybean meal diet [13]. Mwaniki et al. expressed results showing that a percentage inclusion of 7.5% of the diet resulted in similar hen-day egg production and egg mass but a poor feeding conversion rate compared to a corn–soybean meal diet [14].

On the other hand, microalgae have sundry applications including CO_2_ mitigation, biodiesel production, nutraceutical production, pharmaceutical production, and residual water treatment [15,16]. It contains almost all of the essential amino acids in its composition [17], and its protein content is 50–70% in some microalgal species [18] and it contains 10–25% carbohydrates in dry weight [19]; it can also accumulate high quantities of lipids, mainly polyunsaturated fatty acids like eicosapentaenoic acid (EPA) and docosahexaenoic acid (DHA), and some species such as *Nannochloropsis* have an EPA content that is 30–40% of their total fatty acids [20].

In poultry feed, the prevailing species is *Nannochloropsis*. Its utilization at an inclusion rate of 20% has demonstrated an increase in EPA and DHA levels in yolks, accompanied by alterations in yolk color and carotenoid content [21]. Moreover, when it is used as a partial substitute for soybean meal at 8%, it elevates DHA and PUFA n-3 levels without affecting the performance and quality of eggs [22]. In the year 2022, Mens et al. mentioned that its incorporation at inclusion rates of 2% and 3% holds the potential to enhance feed intake and increase EPA and DHA levels in the yolk, while parameters such as yield, nutrient retention, production, and egg quality remained unaffected. But, further exploration is recommended into the health aspects for laying hens [23].

Therefore, the combination of BSFL and MA is a viable substitute to supplant soybean meal in poultry nutrition for laying hens. However, endeavors to enhance productive performance encompassing laying rate, body growth, and egg quality parameters have yielded inconclusive results. It is expected that the addition of the BSFL–MA mix will positively impact the overall performance and production efficacy of laying hens, particularly in terms of growth rate, egg production, and egg quality. The aim of this study is to establish the impact of the addition of a BSFL–MA mix on the overall performance and production efficacy of laying hens.

## 2. Materials and Methods

### 2.1. Animals, Diets, and Management

The feeding trial was carried out at La Bomba, Huimilpan, in Queretaro, Mexico, from November to May. The location features a semi-dry temperate climate with average annual temperatures ranging between 14 and 17.4 °C. Notably, temperatures of 0 °C or below are recorded in February, March, and October, while in May and June, temperatures can rise to 30 °C or higher.

A total of 96 four-week-old Rhode Island Red chicks were acquired from a commercial rearing farm. The chickens underwent a fifteen-day adaptation period before being divided into six pens. During the adaptation period, chicks were fed a standard starter diet and monitored for their health status. Any birds showing signs of illness or weakness were excluded from the study to ensure homogeneity among the experimental units. Each experimental diet was assigned to two replicate pens, with each pen housing 16 chicks, totaling 32 chicks per treatment. Essential facilities such as nest boxes, drinkers, and feeders were provided in all pens. Additionally, each pen featured a 50 cm high exterior door, granting chicks access to a free-range area of 24 m^2^. The free-grazing area was available to the chicks daily between 8:30 and 17:30 h.

The experiment involved developing two experimental feeds: one containing 10% BSFL as the base (Diet A), and another containing 10% BSFL with an additional 2% MA (Diet B). A commercial feed was used as the control (Diet C). The compositions and formulation of the diets for each growth stage are presented in Table 1. The commercial feed (Diet C) was selected to provide a nutritionally adequate baseline for comparison. Its composition, as provided by the manufacturer, included a mix of cereals like maize, sorghum, and wheat. This ensured that differences observed in growth and laying performance could be attributed to the experimental diets.

### 2.2. Laying Performance Determination

Flock monitoring involved body weight (BW) measured in kilograms, the rate of weekly growth (RDG) as calculated in Equation (1), and the laying start age (LSA). Body weight (BW) was recorded on a weekly basis utilizing a digital bascule Digital Salter. The laying start age (LSA) was defined as the point at which the first egg was produced.

(1) $$RDG=\frac{Final\;body\;weight-Initial\;body\;weight}{Number\;of\;weeks}$$

### 2.3. Chemical Analysis of Diets

Samples of diets were finely ground in a pulverizer for analysis of humidity, ash, total fat, calories, carbohydrates, crude protein, and minerals.

Humidity was determined using the gravimetric method according to PROY-NOM-211-SSA-2002. Between 5 and 10 g of the wet sample was weighed in a porcelain capsule at a constant weight. Subsequently, the capsule with the sample was placed in a drying oven at 100 °C for 4 h or until a constant weight was reached.

Ash content was determined using the gravimetric method NMX-F-066-S-1978. Between 3 and 5 g of the sample was placed in a constant-weight capsule. The capsule with the sample was then heated in a muffle at 550 °C for 6 h.

Caloric content was determined using the isoperibolic method with a bomb calorimeter. Between 1 and 1.5 g of the sample was weighed into a metal capsule for calorimetry. The metal capsule, along with a cotton thread, was placed inside the bomb calorimeter, which was filled with oxygen at 400 psi and subsequently introduced into the calorimeter.

Carbohydrates were determined using the Antrona method with a spectrophotometer. A calibration curve with glucose as a standard was prepared. The samples were dried and ground, and the weight of each sample was recorded. The samples were then placed in test tubes, and HCl was added for hydrolysis. The test tubes were placed in a water bath for three hours. Afterward, they were centrifuged, and the supernatant was obtained. The supernatant was placed in a cold bath, and an antrona solution was added. The mixture was brought to a boil for eight minutes, and the concentration was determined at 630 nm using an HACH model spectrophotometer DR6000 (Hach Company, Loveland, CO, USA).

Lipids was determined using the microwave-assisted extraction EPA 3546 (Anton Paar, Graz, Austria) with an extraction using ether 920.39. Crude protein was determined as total nitrogen by the Kjeldahl method.

The minerals Ca, Mg, and Na were determined using an atomic absorption spectrophotometer.

### 2.4. Egg Quality Analysis

Chicken eggs were analyzed to determine the total weight (EW), yolk weight (YW), albumen weight (AW), yolk height (YH), albumen height (AH), equatorial diameter (ED), polar maximum (PM), and color of the yolk (YC) and eggshell (EC). The EW, YW, and AW were determined using an analytical balance PRECISA LS220 (Precisa Garvimetrics AG, Dietikon, Switzerland). The YH and AH were determined separately using a vernier T&O 192-61X-10 (TOEIKOGYO, Japan). The ED and PM were determined using a vernier MITUTOYO CD-6“PSX” (Mitutoyo, Takatsu-ku, Kawasaki, Kanagawa) and with values to determine the shape index in Equation (2). The YC and SC were determined using the CIELAB scale using a colorimeter: KONICA MINOLTA CROMAMETER CR-410 (Konica Minolta, Tokyo, Japan).

(2) $$Index\;shape=\frac{Equatorial\;diameter}{Polar\;maximum}\times 100$$

Samples of yolk and albumen were analyzed in terms of protein and lipids using the methods described previously.

### 2.5. Statistical Analysis

Data for the variables measured were subjected to an analysis of variance (ANOVA) using Statgraphics Centurion XVI (Statgraphics Technologies, The Plains, VA, USA, 2018). Prior to conducting the ANOVA, assumptions of normality were verified using the Shapiro–Wilk test. In the case of significant differences, post hoc comparisons were performed using Tukey’s HSD to determine which groups differed significantly.

The batch of 16 laying hens constituted the experimental unit for the laying performance results (variables measured: rate of weekly growth, survival, laying age, and egg weight). Then, nine eggs represented the experimental unit for the following egg quality parameters: yolk weight, albumen weight, yolk height, equatorial diameter, polar maximum, shape index, and yolk and eggshell color. Finally, a pool of three yolks and three albumen samples of the same treatment group corresponded to the experimental unit for the protein and lipid contents. The results are presented as the mean ± standard error of the means.

## 3. Results

The proximate composition, including moisture, ash, lipids, calories, carbohydrates, protein, and minerals: Ca, Mg, K, and Na, is presented in Table 2 for the growth and development stages. During the growth stage, Diet A exhibited lower levels of carbohydrates, moisture, and Ca compared to Diet B, whereas it displayed higher levels of ash, lipids, energy, protein, Mg, and Na than Diet B. In the development stage, Diet A showed lower levels of moisture, ash, energy, carbohydrates, and Ca than Diet B, while presenting higher levels of lipids, Mg, and Na than Diet B.

In the laying stage (Table 3), Diet A demonstrated lower levels of moisture, carbohydrates, and Ca compared to Diet B, while exhibiting higher levels of ash, energy, and Mg than Diet B. Moreover, the lipid, protein, and Na contents were found to be similar in both feeds.

The results of laying performance are summarized in Table 4. The weekly growth rate showed statistically significant differences among the dietary treatments (*p* < 0.05). Hens fed Diet B exhibited a higher growth rate (0.034 ± 0.001 kg/week) compared to Diet A (0.028 ± 0.001 kg/week) and Diet C (0.026 ± 0.001 kg/week), with Diet B differing significantly from Diet A (*p* < 0.05). This improvement in growth rate may have contributed to the earlier onset of laying observed in hens fed Diet B, who started laying at week 20, followed by Diet A at week 21, and Diet C at week 23.

The survival rate was highest in hens fed Diet C (61.25%), followed by Diet B (59.34%) and Diet A (51.66%). However, differences among the treatments were not statistically significant (*p* > 0.05). Despite this, Diet B, which included black soldier fly larvae and microalgae, still showed promising results in terms of reproductive performance, potentially indicating the benefits of these ingredients for early egg production.

The physical properties of the eggs were evaluated from the onset of laying until week 30. As expected, the egg weight was directly related to body weight and sexual maturity. Hens fed Diet B produced the heaviest eggs, averaging 51.208 ± 0.511 g, which was significantly higher than those from Diet A (49.985 ± 0.511 g; *p* < 0.05) but not significantly different from those fed Diet C (49.998 ± 0.511 g; *p* < 0.05). A similar trend was observed in yolk weight, where eggs produced by hens fed Diet B had the highest yolk mass (14.728 ± 0.530 g), differing significantly from Diet A (13.527 ± 0.530 g) but not from Diet C (14.332 ± 0.530 g). Albumen weight, however, was not significantly different among the three groups (*p* < 0.05).

Eggs from hens fed Diet C had the highest albumen height (4.186 ± 1.394 mm), followed by Diet A (4.105 ± 1.394 mm) and Diet B (3.716 ± 1.394 mm). This suggests that eggs from hens fed Diet C maintained better freshness over time.

Egg shape was also evaluated, as this characteristic is important for both consumer perception and commercial grading. The shape index, which defines a balanced relationship between the equatorial and polar diameters, did not show significant differences among the dietary treatments (*p* < 0.05).

The color of both the yolks and shells was evaluated using the CIE color system. Yolk color a* differed among the groups, with values being highest in the Diet B group (19.017 ± 0.596), followed by the Diet C group (17.522 ± 0.596) and Diet A group (13.343 ± 0.596). Although the Diet B group showed the strongest yolk a* color, differences were not statistically significant compared to the Diet C group (*p* > 0.05). Similarly, yolk lightness (L*) was significantly higher in hens fed Diet A compared to Diet B, whereas yolk b* pigmentation was significantly lower in the Diet B group. These results indicate that diet composition influenced yolk pigmentation.

Yolk protein content was not significantly different among the different diet groups (*p* < 0.05). However, the albumen protein content was significantly higher in hens fed Diet C (75.277 ± 1.382%DM) than Diet A (74.080 ± 1.382%DM), while hens fed Diet B (76.546 ± 1.382%DM) showed the highest value overall. Interestingly, the yolk lipid content was slightly higher in hens fed Diet B (52.434 ± 1.830%DM) compared to Diet A (51.074 ± 1.830%DM) and Diet C (51.770 ± 1.830%DM), although these differences were not significant. The albumen lipid content, however, was significantly higher in hens fed Diet C (0.506 ± 0.128%DM) compared to Diet A (0.479 ± 0.128%DM) and Diet B (0.451 ± 0.128%DM). These results suggest that the inclusion of black soldier fly larvae meal and microalgae can influence nutrient deposition in egg components.

## 4. Discussion

This study evaluated the impact of including BSFL and MA in the diet of laying hens on their productive performance and egg quality. The inclusion of these alternative ingredients in poultry diets is not only innovative but also holds the potential to contribute to the sustainability and improving the efficiency of egg production by offering a high-quality protein source that is rich in essential nutrients such as fatty acids, proteins, and fiber.

The results obtained in this study revealed that the inclusion of BSFL with a mix of microalgae in the diet of RIR laying hens positively affected their weekly growth rate, laying age, and physical quality of their eggs. This finding aligns with the results of previous studies that highlight the effectiveness of black soldier fly larvae as a rich source of proteins and essential amino acids, which could have contributed to a higher growth rate [24]. Moreover, the presence of bioactive compounds in BSFL, such as antimicrobial peptides and chitin, has been associated in the literature with gut health improvements, which may indirectly improve nutrient absorption and overall growth. As for the laying age, birds fed Diet B began egg production one week earlier than those fed Diet C, suggesting a potential role of nutritional composition in the modulation of sexual maturation. The relationship between nutrition and the onset of laying is complex and influenced by multiple factors such as available proteins, fatty acids, and micronutrients, which directly impact reproductive hormones. In particular, microalgae are known for their high omega-3 fatty acid content, which, in previous studies, has been linked to improved reproductive function in laying hens [25]. The inclusion of microalgae in the diet may have had a synergistic effect with the larvae supporting an earlier onset of egg production.

In the laying stage, the use of black soldier fly larvae as a partial substitute for soybean meal did not affect consumption or body weight. These results coincide with those described by Bovera et al. in 2018 and are consistent with the findings of Zhao et al. in 2022, who also reported an increase in body weight with no differences in feed intake and the feed conversion ratio [26,27]. Despite the comparable feed intake, it is important to note that the feed conversion efficiency may have been influenced by the digestibility of BSFL proteins and lipids, although this was not directly assessed in the current study and should be evaluated in future trials.

Regarding survival, birds fed Diet C showed a higher survival rate, although the difference was not statistically significant. This may indicate that Diet C, which may have included other functional ingredients, could have contributed to the better overall health of the birds, reducing stress or promoting better adaptation to the environment. While this finding is not conclusive, it highlights the importance of considering animal welfare in feeding studies, as a well-balanced diet has the potential to improve disease resistance and reduce mortality.

However, given that environmental conditions were not monitored or controlled during the study, external factors such as temperature fluctuations and stocking density could have influenced survival rates, which should be considered in future research.

In the context of egg quality, the inclusion of black soldier fly larvae (BSFL) and microalgae (MA) in the diet was found to improve egg size significantly, with an average weight of 51.936 g, compared to Diets A and C. Kawasaki et al. (2019) also explored the use of a 10% supplementation of BSFL in the diet, observing no effect on the egg laying rate and egg weight, but a notable increase in the richness of the microbiota [28]. Although the yolk weight difference between Diets B, A, and C was not statistically significant, it was observed that hens fed Diet B had a higher yolk weight than those fed Diets A and C. This increase in yolk mass could be due to the abundance of essential nutrients provided by the black soldier fly larvae and microalgae, which are rich in fatty acids and proteins that promote yolk development [29]. Additionally, microalgae, which are rich in bioactive nutrients such as vitamins and minerals, may have also contributed to the improvement in yolk quality. The stability and quality of the albumen in these treatments also align with studies suggesting that balanced diets can maintain the internal quality of eggs, even when unconventional protein sources are included [30].

Egg shape is an important aspect for the industry, as consumers prefer eggs with a normal shape, with a ratio between the equatorial diameter and polar maximum of 3 to 4, resulting in an index between 72 and 76. The results of this study indicate that hens fed Diet B and Diet C produced eggs with shapes closer to the optimal range, while those fed Diet A produced more elongated eggs. The inclusion of larvae and microalgae in Diet B may have impacted the egg structure, as some studies suggest that fatty acids and other bioactive components in these ingredients can influence the egg membrane, affecting its shape [31]. As for egg color, although no significant differences were observed, eggs from Diet B showed greater consistency in their color, both in the shell and yolk. Uniformity in egg color can be a desirable attribute in the industry, as it is indicative of a balanced diet and can influence consumer acceptance. This is particularly important, as consumers associate consistency in color with product quality [32]. Further analysis of the pigment composition in eggs from hens fed Diet B could provide insights into how microalgae influence yolk coloration. For example, the presence of carotenoids in microalgae may have contributed to yolk pigmentation and antioxidant properties, which could be explored further in future studies.

Egg yolk predominantly contains fat and protein as primary macronutrients [33]. Several studies have reported that BSFL meal contains high levels of fatty acids that could be transferred to the yolk [34]. In this study, Diet B, which included BSFL and microalgae, demonstrated a significantly higher lipid content in the egg yolk compared to Diets A and C. This increase in yolk lipids can be attributed to the high lipid profile of BSFL (rich in saturated fatty acids) and lipids of MA (rich in unsaturated fatty acids), particularly linoleic and oleic acid [35]. These fatty acids are known to positively influence yolk quality and could be beneficial for the nutritional profile of eggs. Since these fatty acids have been linked to improved human health, future studies should analyze their specific composition and potential benefits for consumers.

Despite the use of isoproteic experimental diets, the albumen protein content was higher in hens fed Diet B as well. This indicates that the protein from BSFL may be more readily utilized for albumen production, contributing to higher protein levels in the egg white. The differences observed in yolk and albumen protein contents between the diets highlight the complex interactions between dietary components and egg composition. Further research is needed to determine how specific nutrients in BSFL and microalgae contribute to these variations and whether they could be optimized for better egg quality; in addition, further investigation into the bioavailability of these proteins and their potential impact on egg functionality in food applications is warranted.

The results of this study provide evidence that the inclusion of black soldier fly larvae and microalgae in the diet of laying hens can have beneficial effects on egg quality and productive performance. While further research is needed to fully understand the mechanisms behind these effects, the findings suggest that these alternative ingredients may not only improve the sustainability of poultry production but also offer nutritional and commercial advantages. Additionally, future studies should incorporate cost–benefit analyses and environmental control to strengthen the applicability of these findings under commercial conditions.

Furthermore, future research should explore in greater depth the specific fatty acid and protein profiles resulting from the inclusion of BSFL and a BSFL–MA mix, as well as the sensory characteristics of the resulting egg factors that are key for consumer acceptance. Long-term studies are also needed to evaluate the impact on hen health, nutrient digestibility, and metabolic responses. Additionally, considering the observed increase in the yolk lipid content associated with BSFL- and BSFL–MA-containing diets, future studies should assess the use of defatted BSFL to determine the role of dietary fat in performance and product quality, potentially allowing for optimized formulations that balance nutritional benefits with lipid intake.

## 5. Conclusions

The results of this study demonstrated that the inclusion of BSFL and a BSFL–MA mix in the diets of RIR laying hens improved performance parameters, including resulting in an improved weekly body weight gain and earlier laying age.

The inclusion of BSFL in the diet of Rhode Island Red laying hens preserved essential egg quality traits, including the egg weight, yolk and albumen weight, and albumen and yolk height, without altering the egg shape index. While BSFL inclusion modified the yolk color parameters, it did not affect eggshell coloration. In contrast, the BSFL–MA mix improvements were observed with the same physical parameters, but yolk color was further enhanced.

Furthermore, BSFL alone reduced the total lipid content in both the yolk and albumen, while the combination of BSFL and MA modified both lipid and protein composition in these components. These findings indicate that both BSFL and the BSFL–MA mixture contribute positively to the physical and chemical quality of eggs, with the combination producing a more pronounced effect on nutritional composition and visual characteristics.

Additional studies should expand upon these findings by establishing optimal inclusion levels and evaluating the long-term impacts on hen health, nutrient digestibility, and the sensory profile of eggs. Investigating the use of defatted BSFL meal may also help to optimize lipid intake while preserving the nutritional benefits of insect-based feeds.

## Figures and Tables

**Table 1:** Composition of basal diets of laying hens by each stage.

Ingredients (%)	Growth (6–13 Weeks of Age)		Development (13–18 Weeks of Age)		Laying (18 Weeks of Age Onwards)	
	A	B	A	B	A	B
BSFL	10	10	10	10	10	10
MA	0	2	0	2	0	2
Maize	45.4	48.2	38.9	40.6	43.3	38.2
Corn gluten	5.5	5	0	0	0	0
Wheat bran	39.1	34.8	40	40	40	38.1
Soybean meal	0	0	11.1	7.4	6.7	5.9

BSFL: black soldier fly larvae; MA: microalgae; A: diet with 10% BSFL; B: diet with 10% BSFL and 2% MA.

**Table 2:** Comparison of proximate composition of feeds in growth and development stages.

	Growth Stage		Development Stage	
	Diet A	Diet B	Diet A	Diet B
Moisture (%)	14.316	16.74	16.939	20.453
Ash (%)	4.529	4.374	4.325	4.569
Lipids (%)	7.327	6.171	5.865	6.345
Energy Brute (kcal/g)	4.592	4.571	3.263	3.538
Carbohydrates (%)	55.732	60.72	65.535	69.744
Protein (%)	17.541	17.315	17.252	17.106
Ca (mg/g)	0.071	0.093	0.074	0.085
Mg (mg/g)	2.051	1.932	2.14	1.856
Na (mg/g)	0.153	0.146	0.158	0.142

Diet A with 10% BSFL; Diet B with 10% BSFL and 2% MA.

**Table 3:** Comparison of proximate composition of feeds in laying stage.

	Diet A	Diet B	Diet C
Moisture (%)	10.8	15.0	7.267
Ash (%)	4.525	4.369	15.019
Lipids (%)	5.678	5.325	3.251
Energy Brute (kcal/g)	3.263	3.538	3.432
Carbohydrates (%)	65.536	67.589	48.498
Protein (%)	16.563	16.452	16.895
Ca (mg/g)	0.02	0.06	1.104
Mg (mg/g)	2.09	1.98	2.854
Na (mg/g)	0.1	0.1	0.195

Diet A with 10% BSFL; Diet B with 10% BSFL and 2% MA; Diet C, commercial control.

**Table 4:** Egg physical properties evaluated throughout the entire laying-hen cycle using three different diets.

	Diet A	Diet B	Diet C	SEM	*p*-Value
Weekly growth rate (kg/day)	0.028 ^a^	0.034 ^b^	0.026 ^a^	0.001	0.021
Survival (%)	51.666 ^a^	59.340 ^ab^	61.250 ^b^	1.748	0.058
Laying age (week)	21	20	23		
Eggs					
Total weight (g)	49.985 ^a^	51.208 ^b^	49.998 ^a^	0.511	0.155
Yolk weight (g)	13.527 ^a^	14.728 ^a^	14.332 ^b^	0.530	0.283
Albumen weight (g)	23.670 ^a^	22.850 ^a^	23.765 ^a^	1.394	0.878
Yolk height (mm)	11.422 ^a^	12.6256 ^b^	12.9578 ^b^	0.411	0.035
Albumen height (mm)	4.105 ^a^	3.716 ^a^	4.186 ^a^	0.258	0.402
Equatorial diameter	40.314 ^a^	41.155 ^a^	40.828 ^a^	0.505	0.505
Polar maximum	53.037 ^a^	54.027 ^a^	53.630 ^a^	0.713	0.620
Shape index	76.022 ^a^	76.222 ^a^	76.188 ^a^	0.834	0.983
Yolk color l*	84.866 ^b^	80.225 ^a^	83.352 ^ab^	1.535	0.114
Yolk color a	13.343 ^a^	19.017 ^b^	17.522 ^b^	0.596	0.000
Yolk color b	75.122 ^a^	70.846 ^a^	75.527 ^a^	1.828	0.155
Eggshell color l*	75.805 ^a^	73.701 ^a^	74.057 ^a^	2.687	0.840
Eggshell color a	12.501 ^a^	11.826 ^a^	11.560 ^a^	0.753	0.665
Eggshell color b	27.354 ^a^	26.718 ^a^	25.197 ^a^	1.033	0.333
Yolk protein (%DM)	28.32 ^a^	28.185 ^a^	28.893 ^a^	1.310	0.922
Albumen protein (%DM)	74.080 ^a^	76.546 ^a^	75.277 ^a^	1.382	0.561
Yolk lipids (%DM)	51.074 ^a^	52.434 ^a^	51.770 ^a^	1.830	0.873
Albumen lipids (%DM)	0.479 ^a^	0.451 ^a^	0.506 ^a^	0.128	0.954

Data are presented as ordinary mean ± standard error. Different superscripts letters in a row indicate statistically significant differences (ANOVA, *p* < 0.05).

## Data Availability

The original contributions presented in this study are included in the article. Further inquiries can be directed to the corresponding author(s).

## References

[1] Kleyn F., Ciacciariello M. (2021). Future demands of the poultry industry: Will we meet our commitments sustainably in developed and developing economies?. World’s Poult. Sci. J..

[2] Daghir N., Diab-El-Harake M., Kharroubi S. (2021). Poultry production and its effects on food security in the Middle Eastern and North African region. J. Appl. Poult. Res..

[3] Patra A.K. (2021). Advances in Poultry Nutrition Research.

[4] Ravindran V. (2013). Disponibilidad de piensos y nutrición de aves de corral en paises en desarrollo-2. Revisión Desarro. Avícola.

[5] Van der Aar P., Doppenberg J., Kwakernaak C., Burton E., Gatcliffe J., O’Neill H.M., Scholey D. (2016). Which feedstuffs will be used in the future?. Sustainable Poultry Production in Europe.

[6] Mohamed I.A., Emhimad A.A., Ubedullah K., Muhammad Abdul B., Amlan Kumar P. (2021). Nontraditional Feedstuffs as an Alternative in Poultry Feed. Advances in Poultry Nutrition Research.

[7] Lalander C., Diener S., Zurbrugg C., Vinneras B. (2019). Effects of feedstock on larval development and process efficiency in waste treatment with black soldier fly (*Hermetia illucens*). J. Clean. Prod..

[8] Spranghers T., Ottoboni M., Klootwijk C., Ovyn A., Deboosere S., De Meulenaer B., Michiels J., Eeckhout M., De Clercq P., De Smet S. (2017). Nutritional composition of black soldier fly (*Hermetia illucens*) prepupae reared on different organic waste substrates. J. Sci. Food Agric..

[9] Yu G., Chen Y., Yu Z., Cheng P. (2009). Research progress on the larvae and prepupae of black soldier fly *Hermetia illucens* used as animal feedstuff. Chin. Bull. Entomol..

[10] Tran G. (2020). INRA-CIRAD-AFZ.

[11] El-Hack M.A., Abdelnour S.A., Shafi M., Shehata A.M. (2020). Black soldier fly (*Hermetia illucens*) meal as a promising feed ingredient for poultry: A comprehensive review. Agriculture.

[12] Marono S., Loponte R., Lombardi P., Vassalotti G., Pero M., Russo F., Gasco L., Parisi G., Piccolo G., Nizza S. (2017). Productive performance and blood profiles of laying hens fed *Hermetia illucens* larvae meal as total replacement of soybean meal from 24 to 45 weeks of age. Poult. Sci..

[13] Secci G., Bovera F., Nizza S., Baronti N., Gasco L., Conte G., Serra A., Bonelli A., Parisi G. (2018). Quality of eggs from Lohmann Brown Classic laying hens fed black soldier fly meal as substitute for soya bean. Animal.

[14] Mwaniki Z., Shoveller A.K., Huber L.A., Kiarie E.G. (2020). Complete replacement of soybean meal with defatted black soldier fly larvae meal in Shaver White hens feeding program (28–43 wks of age): Impact on egg production, egg quality, organ weight, and apparent retention of components. Poult. Sci..

[15] Mehariya S., Goswami R.K., Karthikeysan O.P., Verma P. (2021). Microalgae for high-value products: A way towards green nutraceutical and pharmaceutical compounds. Chemosphere.

[16] Sajjadi B., Chen W.-Y., Raman A.A.A., Ibrahim S. (2018). Microalgae lipid and biomass for biofuel production: A comprehensive review on lipid enhancement strategies and their effects on fatty acid composition. Renew. Sustain. Energy Rev..

[17] Manor M., Derksen T., Magnuson A., Raza F., Lei X. (2019). Inclusion of Dietary Defatted Microalgae Dose-Dependently Enriches ω-3 Fatty Acids in Egg Yolk and Tissues of Laying Hens. J. Nutr..

[18] Buono S., Langellotti A.L., Martello A., Rinna F., Fogliano V. (2014). Functional ingredients from microalgae. Food Funct..

[19] Chacón-Lee T.L., González-Mariño G.E. (2010). Microalgae for “Healthy” Foods—Possibilities and Challenges. Compr. Rev. Food Sci. Food Saf..

[20] Sun X.M., Ren L.J., Zhao Q.Y., Ji X.J., Huang H. (2018). Microalgae for the production of lipid and carotenoids: A review with focus on stress regulation and adaptation. Biotechnol. Biofuels.

[21] Krienitz L., Wirth M. (2006). The high content of polyunsaturated fatty acids in Nannochloropsis limnetica (Eustigmatophyceae) and its implication for food web interactions, freshwater aquaculture and biotechnology. Limnologica.

[22] Wu Y., Li L., Wen Z., Yan H., Yang P., Tang J., Xie M., Hou S. (2018). Dual functions of eicosapentaenoic acid-rich microalgae: Enrichment of yolk with n-3 polyunsaturated fatty acids and partial replacement for soybean meal in diet of laying hens. Poult. Sci..

[23] Mens A., van Krimpen M., Kar S., Guiscafre F., Sijtsma L. (2022). Enriching table eggs with n-3 polyunsaturated fatty acids through dietary supplementation with the phototrophically grown green algae Nannochloropsis limnetica: Effects of microalgae on nutrient retention, performance, egg characteristics and health parameters. Poult. Sci..

[24] Tahamtani F.M., Ivarsson E., Wiklicky V., Lalander C., Wall H., Rodenburg T.B., Tuyttens F.A.M., Hernandez C.E. (2021). Feeding live Black Soldier Fly larvae (*Hermetia illucens*) to laying hens: Effects on feed consumption, hen health, hen behavior, and egg quality. Poult. Sci..

[25] Ginzberg A., Cohen M., Sod-Moriah U.A., Shany S., Rosenshtrauch A., Arad S. (2000). Chickens fed with biomass of the red microalga Porphyridium sp. have reduced blood cholesterol level and modified fatty acid composition in egg yolk. J. Appl. Phycol..

[26] Bovera F., Loponte R., Pero M., Cutrignelli M.I., Calabro S., Musco N., Vassalotti G., Panettieri V., Lombardi P., Piccolo G. (2018). Laying performance, blood profiles, nutrient digestibility and inner organs traits of hens fed an insect meal from Hermetia illucens larvae. Res. Vet. Sci..

[27] Zhao J., Kawasaki K., Miyawaki H., Hirayasu H., Izumo A., Iwase S.-i., Kasai K. (2022). Egg quality and laying performance of Julia laying hens fed with black soldier fly (*Hermetia illucens*) larvae meal as a long-term substitute for fish meal. Poult. Sci..

[28] Kawasaki K., Hashimoto Y., Hori A., Kawasaki T., Hirayasu H., Iwase S.-i., Hashizume A., Ido A., Miura C., Miura T. (2019). Evaluation of Black Soldier Fly (*Hermetia illucens*) Larvae and Pre-Pupae Raised on Household Organic Waste, as Potential Ingredients for Poultry Feed. Animals.

[29] Makkar H.P.S., Tran G., Heuzé V., Ankers P. (2014). State-of-the-art on use of insects as animal feed. Anim. Feed Sci. Technol..

[30] Haugh R.R. (1937). The Haugh unit for measuring egg quality. U. S. Egg Poult. Mag..

[31] Mine Y. (2008). Egg Bioscience and Biotechnology.

[32] Berkhoff J., Alvarado-Gilis C., Keim J.P., Alcalde J.A., Vargas-Bello-Pérez E., Gandarillas M. (2020). Consumer preferences and sensory characteristics of eggs from family farms. Poult. Sci..

[33] Réhault-Godbert S., Guyot N., Nys Y. (2019). The golden egg: Nutritional value, bioactivities, and emerging benefits for human health. Nutrients.

[34] Heuel M., Sandrock C., Leiber F., Mathys A., Gold M., Zurbrügg C., Gangnat I.D.M., Kreuzer M., Terranova M. (2021). Black soldier fly larvae meal and fat can completely replace soybean cake and oil in diets for laying hens. Poult. Sci..

[35] Ohse S., Derner R.B., Ozório R.Á., Corrêa R.G., Furlong E.B., Cunha P.C.R. (2015). Lipid content and fatty acid profiles in ten species of microalgae. Idesia.

